# Coasting related to taxane-induced peripheral neuropathy in patients with breast cancer: a systematic review

**DOI:** 10.2340/1651-226X.2025.42109

**Published:** 2025-01-15

**Authors:** Freja L. Kruse, Margrethe B. Bille, Maria E. Lendorf, Susan Vaabengaard, Steffen Birk

**Affiliations:** a Department of Oncology, Rigshospitalet, Copenhagen, Denmark; b Department of Clinical Neurophysiology, Rigshospitalet, Copenhagen, Denmark; c Department of Oncology and Palliative Care, Nordsjællands Hospital, Hillerød, Denmark

**Keywords:** CIPN, TIPN, neurotoxicity, delayed onset, chemotherapy, late effects

## Abstract

**Background and purpose:**

Chemotherapy-induced peripheral neuropathy (CIPN) is a common dose limiting adverse effect that may be transient or become persistent after the treatment ended. The taxane paclitaxel induces CIPN in 57–83% of patients treated. The neuropathy may debut or progress after the end of treatment (EOT), known as coasting, but little is known about the incidence of this phenomenon. The aim of this review is to examine the incidence and severity of coasting in CIPN in patients with breast cancer.

**Patient/material and methods:**

MEDLINE, Embase, clinicaltrials.gov, and medrivx.org were searched using terms related to taxanes, adverse effects, and breast cancer. Studies had to have a follow-up time of at least 3 months after EOT and patients had to have received taxanes in monotherapy. Additionally, studies had to be longitudinal and describe the neuropathy assessment method and timing.

**Results:**

A total of 17 studies met the eligibility criteria, with 4,265 participants summarized. Of these, one study reported coasting events in 14.3% (*n* = 4) of patients. Eight studies reported no coasting events and eight were unclear.

**Interpretation:**

Few studies reported on coasting in CIPN. There may be several reasons for this, including the timing and choice of assessment methods, confounding factors, and the possible rarity of the phenomenon. More information is needed about coasting in CIPN to better characterize the neuropathies, guide patient and doctor decisions, and aid in the development of interventions toward CIPN.

## Background

The increasing number of cancer survivors highlights the need to focus on the acute and chronic toxic effects from curative treatment. Chemotherapy-induced peripheral neuropathy (CIPN) is a debilitating, dose-limiting, and common adverse effect from numerous chemotherapies, including platinum compounds, taxanes, vinca alkaloids, bortezomib, and thalidomide [1–3]. Large clinical trials have confirmed a survival benefit from taxanes, and taxane-based therapies are integral to the treatment of breast cancer [4–6]. For breast cancer patients, CIPN can affect the quality of life [7, 8]. The degree of neuropathy depends on several factors such as cumulative dose and duration of therapy [3]. CIPN commonly presents as a distal, symmetric, mainly sensory neuropathy, with a stocking-and-glove-distribution [9]. No recognized strategy exists for CIPN prevention and pharmacological options to manage established CIPN are limited [3, 10]. CIPN can result in dose reductions and treatment terminations, potentially compromising the efficacy of treatment and patient survival [1, 7, 11]. Neuropathy can be long lasting and may even debut or worsen after treatment [12, 13]. This phenomenon is called coasting and is quite frequently observed in platinum drugs and vinca alkaloids [10, 14]; however, it is less clear how often it occurs in patients treated with taxanes [10, 15–17]. Limited knowledge exists on the prevalence, persistence, and severity of CIPN beyond the acute phase of cancer treatment.

It is important to know the incidence of coasting in CIPN to better inform patient decisions. In addition, characterization of incidence of possible variants in the debut and course of CIPN supplies a better foundation for assessing the safety and efficacy of interventions toward CIPN. For example, a large clinical trial that observed a possible benefit of local cooling therapy for CIPN prevention observed coasting in 10% (*n* = 76). Though in this trial it did not lead to persistent neuropathy [17]. Without knowing the incidence of coasting it is difficult to know if the coasting was exacerbated by the cooling therapy.

After searching the literature and to the best of our knowledge, there is no well-established definition of coasting. We define coasting as CIPN that debuts or progresses after the end of treatment (EOT). Furthermore, we define EOT as 3 weeks or more after the last administered dose of chemotherapy, as debut or worsening before this cutoff cannot be separated from the effects of the last dose of chemotherapy.

The aim of this study is to identify clinical studies that should be able to observe coasting in patients receiving taxanes, defined by a sufficient follow-up period and assessment methods. Through analyzing these studies, it may be possible to assess the incidence of coasting in CIPN related to taxanes.

## Materials and methods

### Protocol

A protocol for the review was created using PRISMA-P guidelines (Supplementary C and D).

### Eligibility criteria

The eligibility criteria were chosen to minimize confounding factors while making it applicable to the clinical setting. Included studies had to have at least one group with taxanes in monotherapy, no preventive interventions towards CIPN, and a well-described incidence of CIPN. It was allowed for chemotherapy, that is not neurotoxic, to be given sequentially with taxane therapy. Anti-HER-2 (human epidermal growth factor receptor 2) directed treatment, endocrine therapy, and radiation, could be given concurrent with taxane treatment. As this is an attempt at establishing the incidence of coasting related to taxane therapy, not focusing on cases but how many in a population experiences coasting, studies with less than 10 patients were excluded. The follow-up period had to be at least 3 months after EOT. This was chosen as coasting is reported to occur around 3 months after EOT in platinum compounds [10]. To our knowledge, cancer type is not associated with risk of CIPN [18, 19]; however, the scope of this review was restricted to patients with breast cancer, to keep the number of relevant studies at a manageable level and for homogeneity of the studies. To ensure that studies would be able to detect coasting if present, the assessment method and timepoints for assessment and severity had to be sufficiently described.

### Search and information sources

A combination of databases was searched during February and March 2021: PubMed (MEDLINE, 1966–2021), Embase (OVID, 1974–2021), clinicaltrials.gov, and medrivx.org. A detailed description of the search methods can be found in the supplementary material (Supplementary A).

An additional search of MEDLINE in March 2021 was conducted to ensure that the relevant studies with platinum mentioned would be found. Additional hand searches were performed in central reviews [15, 18, 20–22]. The searches were conducted under the guidance of an Information Specialist. The search was repeated in October 2022, to find studies published since the original search.

### Study selection

The abstracts and full texts were screened by one reviewer (FLK) with supervision of a second reviewer (SV). For studies difficult to assess, both reviewers contributed to the screening. The studies were managed using COVIDENCE software [23].

### Data items

The information extracted from the included trials were: Trial and patient characteristics with a focus on primary and secondary outcomes, interventions towards CIPN, comorbidities, and peripheral neuropathy at baseline; chemotherapy regiment and additional anti-neoplastic treatment; assessment method for neuropathy; incidence of neuropathy and incidence, course, and grade of coasting.

Missing information was recorded as *Not Available* (NA) if no information was given and *Unclear*, if too much had to be assumed. If the incidences of neuropathies were reported separately for several eligible groups in the same study, it was summarized.

### Critical appraisal

The quality of the studies was assessed using the Joanna Briggs Institute Critical Appraisal Checklist for Studies Reporting Prevalence Data [24].

## Results

After removing duplicates, 4,344 studies were screened by title and abstract for relevance. A total of 721 went on to full-text screening, of which 15 were eligible. An additional two studies were identified through hand searches, resulting in a total of 17 studies included for analysis. Most of the studies not included were due to the follow-up time being too short (*n* = 244) or not specifying when the time of maximal CIPN occurred (*n* = 221) See PRISMA tabel in supplementary material Appendix B.

Tabels 1-3 sum up characteristics of the included studies. Overall, the samples were appropriate and matching the general characteristics of patients with breast cancer. However, some important issues need mentioning. The studies were not very ethnically diverse with the majority primarily Caucasian. Additionally, some studies had restrictions, that make them heterogeneous and difficult to compare. One study excluded patients >70 years of age and had only 25% of patients over 50-years-old. Patients with psychiatric comorbidities and shift work were also excluded from this study, which may cause bias [25]. Pabst et al. who reported coasting, had a sample of elderly patients aged 65 years and older with a median of 70.5 years, which may have affected the outcome [26]. Most of the studies had an acceptable response rate from participants [26–34]. A detailed critical appraisal can be found in the supplementary material (Supplementary F).

**Table 1:** Study characteristics.

Author + year	Published?	Patients included overall	Patients included in taxane in monotherapy	Patients analyzed or taxane in monotherapy	Study type	Study design	Primary endpoint(s)	Secondary endpoint(s)
*Bandos 2018*	Yes	2,051	684	684	RCT	Prospective	Prevalence and severity of PN over time and impact on QOL and factors associated with long term PN	NA
*Greenlee 2017*	Yes	4,505	1,237	771	Cohort	Prospective	Lifestyle factors in CIPN	NA
*Hershman 2018*	Yes	437	218	173	RCT	Prospective	Acetyl-L-carnitine vs placebo for CIPN prevention	Long-term CIPN and phenotype
*Hershman 2011*	Yes	50	50	50	Cohort	Prospective	Natural history and long term prevalence and severity of CIPN assessed by FACT-Ntx	Self-reported measures for CIPN compared with QST
*Lee 2018*	Yes	143	143	111	Observa-tional	Prospective	Prevalence and risk factors for CIPN	NA
*Martin 2008*	Yes	1,248	614	594	RCT	Prospective	FEC vs FEC-PTX	Associations between various molecular characteristics and response to taxane treatment
*Ng 2020*	Yes	46	23	17	RCT	Prospective	Cryotherapy intervention for CIPN (PNQ grade C-E 1–2 weeks after treatment	PNQ grade C-E 3–9 months post paclitaxel
*Nitz 2014*	Yes	2,012	1,950	978	RCT	Prospective	Comparing taxane vs. Non-taxane regimes	Retrospective investigation of potential immuno-histological predictors of taxane outcome
*Pabst 2020*	Yes	320	434	213	Cohort	Retrospective	Frequency of persistent grade 2 and 3 CIPN. Identification of risk factors for clinically meaningful (grade 2 or higher) CIPN	NA
*Pace 2007*	Yes	17	17	11	Cohort	Prospective	Incidence of PIPN	NA
*Pachman 2017*	Yes	45	23	23	RCT	Prospective	Pilot minocycline for pre-vention of P-APS and PIPN	NA
*Ruddy 2019*	Yes	46	23	20	RCT	Prospective	Pilot investigating cryotherapy for prevention of CIPN	Cryotherapy tolerability
*Shinde 2016*	Yes	46	23	22	RCT	Prospective	Pregabalin intervention for prevention of P-APS and PIPN	NA
*Shimozuma 2012*	Yes	300	300	260	RCT	Prospective	Presence of non-inferiority of single agent taxane vs. AC followed by taxane in terms of disease free survival	Tolerability of taxane regiment and HRQOL Time course and severity of patient reported CIPN during treatment and 1 year post treatment
*Tanabe 2013*	Yes	225	212	212	Cohort	Retrospective	Determine duration of PIPN and identify factors predicting severe or persistent PN	NA
*Thornton 2008*	Yes	227	55	55	Case control	Prospective	Short-term, moderate-term and long-term toxicity and QOL of patients receiving taxanes	NA
*Timmins 2021*	Yes	83	83	71	Cohort	Prospective	PN development and deficits in patients with BC during weekly PTX	Impact of dose reductions on post treatment clinical and patient reported PN outcomes

PN: peripheral neuropathy; QOL: Quality of Life; RCT: Randomized Controlled Trial; CIPN: Chemotherapy-induced Peripheral Neuropathy; QST: Quantitative Sensory Testing; FEC: fluouracil + epirubicin + cyclophosphamide; PTX: paclitaxel; PNQ: Patient Neurotoxicity Questionnaire; PIPN: Paclitaxel Induced Peripheral Neuropathy; P-APS: Paclitaxel – Acute Pain Syndrome, AC: antracycline + cyclophosphamide; HRQOL: Health Related Quality of Life; BC: Breast Cancer: C: cycle, a cycle is usually 21–28 days; NA: non answer.

**Table 2:** Patient characteristics.

Author + year	Age	Sex	Diagnosis	Comorbidities	PN at baseline	Type of taxane	Other chemotherapy	Other treatment allowed if indicated	Taxane Schedule (dose: mg/m^2^)	Cumulative dose (mg/m^2^)
*Bandos 2018*	< 50 (341), > 50 (343)	F	EBC	NA	15.8% (*n* = 108)	DOC	AC	EN, RT	60–75triW/4C	400
*Greenlee 2017*	54 (SD10.6)	F	EBC	Obesity (65. 6%),	NA	DOC, PTX	NA	NA	NA	NA
*Hershman 2018*	51.9 (SD 10.9)	F	EBC	Prior PN or Diabetes excluded	No	PTX, DOC	NA	Placebo (cellulose)	80w/12C, 175biW/4C (PTX) 75triW/4C, 75triW/6C (DOC)	NA
*Hershman 2011*	48 (28–78)	F	EBC	NA	NA	PTX	AC	TX, EN	175biW/4c, 175biW/6c, 175W/12c	NA
*Lee 2018*	44 (SD 7.5)	F	EBC	Unclear^b^	Unclear^b^	DOC	AC	TX, EN, RT	4c	NA
*Martin 2008*	50 (23–76)	F	EBC	Serious medical condition other than BC excluded	No	PTX	FEC	EN, RT	100w/8W	NA
*Ng 2020*	53.6 (SD 7.6)	F	EBC	Diabetes (*n* = 1)	No	PTX	AC	EN, TX, RT	80w/12c	929.4 (SD 28.6)
*Nitz 2014*	51.9	F	BC	NA	No	DOC	EC	NA	100triw/4c	NA
*Pabst 2020*	70.5 (median)	F	EBC	Cardiovascular (74%), Diabetes (21.5%), Dyslipidemia (48.7%)	No	PTX, DOC	NA	NA	NA	NA
*Pace 2007*	55 (W), 57 (triW)	F	BC	NA	No	PTX	NA	NA	80w/24w, 175triW/24w	892.7 (SD175.8) (12w), 1744 (SD 279) (24w)
*Pachman 2017*	54.9 (SD 10.9)	F	BC	NA	NA	PTX	NA	Placebo, TX	80w/12w	NA
*Ruddy 2019*	55 (49–66)	F	EBC	Diabetes (*n* = 1), Prior PN, fibromyalgia, Raynaud or cryoglobulinemia excluded	No	PTX	No	TX, AC	80w/12w	NA
*Shinde 2016*	53.7 (SD 13.7)	F	BC	NA	No	PTX	No	Placebo, TX, EN	80w/12w	NA
*Shimozuma 2012*	50–54	F	EBC	Diabetes (*n* = 2)	Yes, not severe (*n* = 1)	PTX, DOC	AC	NA	175triW/4c (PTX), 75triW/4c (DOC), 175triW/8c (PTX), 75triW/8c (DOC)	NA
*Tanabe 2013*	53 (22–70)	F/M	BC	Diabetes (*n* = 18)	No severe PN	PTX	AC	EN, TX, RT	80w/4c, 175triW/4c	Unclear
*Thornton 2008*	49 (SD 9.6)	F	BC	NA	NA	PTX, DOC	AC	NA	NA	NA
*Timmins 2021*	52.7 (SD 1.2)	F	BC	Diabetes (*n* = 7)	No	PTX	No	TX	80w/12w	861.8 (SD 15.9)

EBC: Early Breast Cancer = invasive, operable, and locally advanced; DOC: docetaxel, AC: antracycline + cyclophosphamide; EN: endocrine therapy; RT: radiotherapy; PTX: paclitaxel; TX: trastuzumab (anti- HER2 treatment); FEC: fluouracil + epirubicin + cyclophosphamide; BC: Breast Cancer; M: male; F: female; NA: non answer; SD: standard deviation; W: weeks; biW: biweekly; triW: triweekly.

**Table 3:** Peripheral neuropathy and coasting.

Author + year	Assessment methods	Timing for CIPN assessment during treatment	Timing for CIPN assessment during follow-up	Highest incidence of PN during treatment-EOT	Incidence of persistent PN 6m-1y	Incidence of persistent PN >2y	Peak incidence of CIPN after EOT?	Patients w. coasting	Peak grade of PN coasting	Time of coasting debut	Time of coasting resolved
*Bandos 2018*	BCPTsc	Baseline (before AC), d1c4 (before taxane)	6m, 12m, 18m, 24m	NA	68.2% (6m)	41.9% (2y)	Unclear^f^	U	NA	NA	NA
*Greenlee 2017*	FACT/GOG-ntx	Baseline (270 had started taxane before baseline)	6m, 24m	NA	28.1% (*n* = 217, 6m)	20.4% (*n* = 111, 2y)	Unclear^f^	U	NA	NA	NA
*Hershman 2018*	FACT/GOG-ntx	Baseline, W12, W24 (EOT)	W36, W52, W104	28% (5p, FACT-Ntx, EOT)	Unclear	34.4% (5p FACT-Ntx, 2y)	Unclear	Unclear	NA	NA	NA
*Hershman 2011*	FACT/GOG-ntx , VT, TT, CTCAE3	Baseline, 2w post last treatment	3m, 6m, 9m, 12m	80% (CTCAE)	67% (12m)	NA	No	0	Irrelevant	Irrelevant	Irrelevant
*Lee 2018*	Non validated numeric scale^a^	Baseline, Last cycle	8m	45% (EOT)	18.9% (8m)	NA	No	0	Irrelevant	Irrelevant	Irrelevant
*Martin 2008*	CTCAE1	Day21/C	3M year 1 + 2, 6M year 3–5, yearly^h^	25.9% (grade 2–3)	NA	NA	No	0	Irrelevant	Irrelevant	Irrelevant
*Ng 2020*	PNQ, NCS (*n* = 12), SSR	Baseline, EOT (1–2 weeks post treatment)	3m (%NCS), 6m (%NCS), 9m	23.5% (PNQ C-E, EOT)	41.2% (PNQ C-E, 6m and 9m)	NA	Unclear	Unclear	NA	NA	NA
*Nitz 2014*	CTCAE2	Each cycle	Every 3m (y2), 6m (till study end)	NA	14.20%	3.2% (2y)	NA	NA	NA	NA	NA
*Pabst 2020*	CTCAE	EOT	2y	70.50%	NA	46.7% (2y)	No	4	3	Unclear	Unclear
*Pace 2007*	TNS	Baseline, 12W, 24W	Mean 6m (interval 4–17m)	96% (*n* = 13)	PN reported as mean score	PN reported as mean score	No	0	Irrelevant	Irrelevant	Irrelevant
*Ruddy 2019*	EORTC-CIPN20, CTCAE	Baseline, weekly	Monthly for 6m	NA*h*	NA	NA	NA	NA	Irrelevant	Irrelevant	Irrelevant
*Pachman 2017*	EORTC-CIPN20, CTCAE4	Baseline, prior to each dose	1m, 2m, 3m, 4m, 5m, 6m	PN reported as mean score	PN reported as mean score	PN reported as mean score	Unclear^d^	NA	NA	NA	NA
*Shinde 2016*	EORTC-CIPN20	Baseline, prior to each cycle	Every 1m (for 6m)	PN reported as mean score	PN reported as mean score	PN reported as mean score	PN reported as mean score	0	Irrelevant	Irrelevant	Irrelevant
*Shimozuma 2012*	CTCAE2, PNQ, FACT/GOG-ntx	Baseline c3, c5, c7	7m, 1y	PTX 12.3%, DOC 14.9%, (PNQ D-E (severe), c7)	PTX 7.9%, DOC 21.2 (PNQ D-E (severe), 7m)	NA	Unclear	NA	NA	NA	NA
*Tanabe 2013*	CTCAE3	Unclear^g^	Unclear^g^	97%	64% (1y)	41% (3y)	No	0	Irrelevant	Irrelevant	Irrelevant
*Thornton 2008*	Non-validated scale^c^	Baseline, 4m, 8m, 12m	Ever 4m (1y), every 6m (y2–5)	Unclear^e^	Unclear^e^	Unclear^e^	No	0	Irrelevant	Irrelevant	Irrelevant
*Timmins 2021*	FACT/GOG-ntx , TNSc, NCS	Baseline, w6, w12	3m, 6m, 12m	85.5% (symptoms hands) (EOT)	55.9% (symptoms, feet, 6m)	NA	No	0	Irrelevant	Irrelevant	Irrelevant

a , Symptoms of numbness or tingling rated from 0 to 10;

^b^, Patients with significant other medical conditions were excluded;

c , Patients were asked to rate symptoms of paresthesia, numbness, motor weakness and incontinence from 0 to 4 equal to the CTCAE;

d , EORTC mean did show both increasing and decreasing mean symptoms during follow-up on different symptoms;

e , Reported as different symptoms of CIPN, overall incidence unclear;

f , Peak incidence at 6m, no assessment between baseline and 6m;

g , Scoring based on oncologist notes during/after treatment;

h , Results were estimated as mean scores in the EORTC-CIPN20 for neuropathy for the group and not given as an incidence.

CIPN: Chemotherapy-induced Peripheral Neuropathy; PN: peripheral neuropathy; BCPTsc: Breast Cancer Prevention Trial symptom checklist; FACT/GOG-ntx: Functional Assessment of Cancer Therapy/Gynecologic Oncology Group – Neurotoxicity, (5p = 5-point chance from baseline); EOT: end of treatment; CTCAE: National Cancer Institute Common Terminology Criteria of Adverse Events; PNQ: Patient Neurotoxicity Questionnaire; NCS: nerve conduction studies; TNSc: Clinical version of the Total Neuropathy Score; EORTC-CIPN20: European Organization of Research and Treatment of Cancer – Chemotherapy-induced Peripheral Neuropathy 20; CTCAE: National Cancer Institute Common Terminology Criteria of Adverse Events; C: cycle, a cycle is usually 21-28 days; D: day; NA: non answer; SSR: Sympathetic skin response; TNS: Total Neuropathy Score; TT: tactile threshold (QST); VT: vibration threshold (QST); W: weeks; m: months.

One study by Pabst et al. reported coasting [26]. In this report with small patient numbers, four patients (14.3%) had a worsening in neuropathy symptoms from a CTCAE grade 1–2 at the EOT to a CTCAE grade 3 after treatment ended during the 2-year follow-up. It was not reported when the symptoms worsened or if the patients had recovered during the 2 years. The study also stated that no patient developed new neurologic symptoms after the EOT.

Eight studies reported no coasting phenomenon or that all neuropathies resolved after EOT [25, 30, 31, 33, 35–38].

Eight studies were unclear about coasting. This was because the results of the long-term follow up were unclear [27, 32, 39] or there was no assessment between baseline and 6 months follow-up [34, 40]. Another four studies only reported mean peripheral neuropathy scores for all patients [29, 30, 36, 41].

All included studies were in the adjuvant or neoadjuvant setting.

Meta-analysis was deemed inappropriate, as the included studies were too different in design and outcome measures and too few actively reported on the occurrence of coasting or lack thereof.

## Discussion

The evident scarcity in the reporting of coasting may rely on several factors. In the following paragraphs assessment method, timing, and missing information will be discussed.

The reporting of coasting and the characterization of the neuropathies are affected by the method of assessment [49–51]. Several of the included studies utilized multiple assessment methods, either different patient reported outcomes (PRO’s) (FACT-Ntx, PNQ, EORTC-CIPN20) and/or clinician reported outcomes (CRO’s) (NCI-CTC) [29, 32, 34, 40, 41] or combining PRO’s with objective or paraclinical measures [33, 38, 39]. Nine studies used only one assessment method [25–28, 30, 31, 34–37, 40]. Two studies utilized a non-validated assessment method [25, 31]. One asked only about symptoms in the last 24 hours [25] and the other was described similar to the CTCAE but was not specific to hands and feet and therefore the items on paresthesia were not included due to being confused with operative complications [31].

A combination of modalities may increase the chance of observing more subtle changes during the neuropathy. Measures such as neurological examinations, quantitative sensory testing (QST) and nerve conduction studies (NCS) may fail to capture symptoms of neuropathy and changes within the normal reference values, if there are no baseline values. However, subjective measures may be subject to many confounding factors, and fail to identify signs of neuropathy, without symptoms [49, 52–53]. In Bandos et al. it is mentioned that PRO’s may not specifically ask if neuropathies have become worse or better [40] – which may affect the reporting of changes. A review of NCS in CIPN mentioned that coasting in other CIPN drugs is associated with a further reduction or loss of Sensory Nerve Action Potential (SNAP) and/or Compound Muscle Action potential (CMAP) and that there may be both symptoms of CIPN without changes in SNAP and/or CMAP and vice versa [13]. The studies included which utilized NCS [33, 39] were either unclear or did not report coasting and had relatively small sample sizes. They did not report the incidence of neuropathy diagnosed with NCS, but rather a mean change from baseline as a group.

Furthermore, difficulty in distinguishing symptoms of neuropathy from other side effects of chemotherapy may lead to both over and underreporting. It was reported in a qualitative study that too simplistic descriptions or analogies could make it difficult for some patients to recognize CIPN symptoms [42]. Moreover, other adverse effects may be prioritized. A study showed that most patients (27%) may wait to report less acute adverse effects such as tingling and numbness to the next appointment, though very few would do nothing (3.9%) [37]. Several factors, including perhaps the gradual onset of neuropathies, may affect the timelines of the reporting and recall bias or untimely registration may be an issue [54]. Other factors associated with the reporting of adverse effects may be the patient’s prior knowledge about the neuropathies. A qualitative study showed that among other enablers of CIPN reporting, was knowledge of long-term consequences of CIPN or family members knowledge of CIPN, while a lack of knowledge about CIPN could be a deterrent to disclosure [42]. Due to the few studies reporting on coasting at all, it is not possible to identify from this review if one method is superior in identifying coasting.

Only five of the included studies comprised an EOT assessment [26, 27, 38, 39, 41]. One study reported coasting, three were unclear, and one did not report coasting at all.

The prevalence of coasting in CIPN may depend on the pathophysiology. The mechanism behind CIPN development is not fully elucidated and several theories exist [15]. As described earlier, coasting is more well-known with platinum compounds [10]. Excitability studies of platinum induced peripheral neuropathy show an early excitability change with a delayed axonal degeneration [43], the study suggests that channel dysfunction is behind delayed axonal degeneration. Similarly, other studies on paclitaxel among other drugs in animal models suggest that changes in the Ca^2+^ homeostasis cause inflammatory responses and/or an effect on the mitochondria [44, 45]. In these studies it is suggested that insufficient antioxidant levels allow more radical oxidative stress (ROS) development, causing a delayed response to the toxic agent, which could be behind the coasting phenomenon. Though this warrants studies to elaborate and confirm [46]. Excitability studies of paclitaxel-treated patients did show early and prolonged sensory dysfunction, but no channel dysfunction. However, channel dysfunction may not be found in such studies, if hid by axonal dysfunction [47].

Other reasons for missing information on coasting, may be that coasting is a relative rare event in taxane treatment [17]. Added together for all studies that had EOT assessments and were clear on presence of coasting, 4/1,339 patients presented with the phenomenon. In the included study reporting coasting it was 14.3% (*n* = 4) [26] and in another not included study, where patients had received cryotherapy, it was 10% (*n* = 76) [17]. An appropriate sample size for observing coasting if the incidence Is 10% can be calculated by using the method recommended by the Joanne Briggs Institute [24]. (see supplementary E). A sample size of 138 should be sufficient to detect coasting if it occurs in 10% of the patients. A total of 9/16 studies had >138 patients included [26–28, 32, 34, 35, 37, 40, 41]. Of these, one observed coasting [26], two [32, 35] did not observe coasting, and the remaining studies were unclear [26, 32, 35, 41]. So, there is not sufficient evidence to conclude how rare coasting may be in CIPN, and being rare alone, does not seem to explain the missing information.

This review has the following limitations. The screening and analysis of the studies included was done by only one reviewer, under supervision. The searches were done in MEDLINE using the Boolean term ‘NOT’ for excluding platinum compounds, and it was attempted to compensate for this with an additional search. But in general, it increases the risk of losing relevant studies. Additionally, some studies were unavailable for full text assessment, which introduces bias. Also limitations are introduced by the eligibility criteria. To make the findings applicable to the clinical setting and most studies using taxanes, it was allowed for the patients to have received other treatments and anti-neoplastic treatment sequentially. This could be an important confounding factor. Finally, a major issue in establishing the incidence of coasting was the amount of missing information: In several studies, it was not reported when the maximum grade of neurotoxicity occurred or if it was assessed after EOT. This could mean that symptoms of peripheral neuropathy only occurred during treatment in these studies, but the missing information renders this to guess work, and it was chosen not to include these studies. It was not within the resources of this review to obtain data or protocol from all these studies. Lastly, only patients with breast cancer receiving taxanes in monotherapy were included. This means that there may be information on coasting in patients receiving taxane therapy for other solid tumors or in combination with non-neurotoxic chemotherapy.

In conclusion, few studies reported on coasting in CIPN. It remains unclear as to how often coasting occurs in patients with breast cancer receiving taxanes in monotherapy. This may be due to several factors. Among these are inconsistencies in assessment, lack of studies with long-term follow up, and possibly the phenomenon being either very rare or underreported. Attention to coasting may be beneficial when designing future studies of CIPN. This will help patients and clinicians, when deciding how to act on neuropathies arising during the treatment. Also, this would aid to better prepare the patients for what to expect. Likewise, research on the mechanisms behind the neuropathy may benefit from knowledge on how it develops and may give a better comparison when evaluating efficacy and safety of preventive interventions towards CIPN.

## Author contributions

FK developed the research question and search string, performed the screening, analysis, and manuscript development and revision.

SV was the second reviewer on difficult assessments and partook in manuscript revision.

MEL, MBB, and SB partook in development of research question and supervised development of study protocol, screening, and analysis and manuscript development and revision.

## Supplementary Material

Coasting related to taxane-induced peripheral neuropathy in patients with breast cancer: a systematic review

## Data Availability

Articles assessed in the study were kept in covidence software and it is not possible to make them publicly available.
