# Supplementary material for: Coasting related to taxane-induced peripheral neuropathy in patients with breast cancer: a systematic review
Source: Acta Oncol. 2025 Jan 15;64:42109. doi: 10.2340/1651-226X.2025.42109 (PMC11748177; doi:10.2340/1651-226X.2025.42109)
Supplement: Coasting related to taxane-induced peripheral neuropathy in patients with breast cancer: a systematic review [file AO-64-42109-s1.pdf]

Supplementary material has been published as submitted. It has not been copyedited, or typeset by Acta Oncologica

## Appendix A - Searches

| MEDLINE (PubMed 1966-2021) | |
|---|---|
| **Search string** | **Additional information** |
| (((((( "Taxoids/adverse effects"[Mesh] OR "Taxoids/toxicity"[Mesh] )) AND "Breast Neoplasms"[Mesh]) NOT "Oxaliplatin"[Mesh]) NOT "Carboplatin"[Mesh]) NOT "Platinum Compounds"[Mesh]) NOT "Cisplatin"[Mesh] | Filter: English<br>Hits: 1527<br>Dato: 23/02/2021<br>New search: 21/10/22<br>Hits: 1673 |
| (((((((paclitaxel[Title/Abstract]) OR (docetaxel[Title/Abstract])) OR (taxan*[Title/Abstract])) OR (taxotere[Title/Abstract])) OR (taxol[Title/Abstract])) AND ((breast cancer[Title/Abstract]) OR (cancer mammae[Title/Abstract]))) AND ((((adverse effect*[Title/Abstract]) OR (toxic[Title/Abstract])) OR (toxicity[Title/Abstract])) OR (tolerability[Title/Abstract]))) NOT (((oxaliplatin[Title/Abstract]) OR (cisplatin[Title/Abstract])) OR (carboplatin[Title/Abstract])) | Filter: English, last 5 years<br>Hits: 676<br>Dato: 23/02/2021<br>New search: 21/10/22<br>Hits: 699 |
| ((( "Taxoids/adverse effects"[Mesh] OR "Taxoids/toxicity"[Mesh] )) AND "Peripheral Nervous System Diseases"[Mesh]) AND ("Breast Neoplasms"[Mesh]) | Dato: 20/3/21*<br>Hits 151<br>New search: 21/10/22<br>Hits: 193 |
| ((((((((paclitaxel[Title/Abstract]) OR (docetaxel[Title/Abstract])) OR (taxol[Title/Abstract])) OR (abraxane[Title/Abstract])) OR (taxotere[Title/Abstract])) AND (((((neuropathy[Title/Abstract]) OR (neurotoxicity[Title/Abstract])) OR (neurotoxic[Title/Abstract])) OR (neuropath*[Title/Abstract])) OR (paresthesia[Title/Abstract]))) AND ((breast cancer[Title/Abstract]) OR (mamma[Title/Abstract]))) AND ((((TIPN[Title/Abstract]) OR (CIPN[Title/Abstract])) OR (DIPN[Title/Abstract])) OR (PIPN[Title/Abstract])) | Dato: 20/3/21*<br>Hits 65<br>New search: 21/10/22<br>Hits: 86 |
| **37 additional studies for screening after dublicates removed and led to another 21 relevant studies on title/abstract screening.* | |

| OVID (Embase 1974-2021) | |
|---|---|
| **Search string** | **Additional information** |
| 1. exp paclitaxel/ae, to [Adverse Drug Reaction, Drug Toxicity]<br>2. exp docetaxel/ae, to [Adverse Drug Reaction, Drug Toxicity]<br>3. exp breast cancer/<br>4. 1 or 2<br>5. 3 and 4<br>6. limit 5 to (english language and exclude medline journals) | Hits: 789<br>Dato: 26/02/21<br><br>New search 21/10/22:<br>Hits 1575<br>824 dublicates removed |

| medrivx.com | |
|---|---|
| **Search string** | **Additional information** |
| full text or abstract or title "paclitaxel docetaxel taxane taxanes taxol taxotere" (match whole any) | Filters: Topic: Oncology, Neurology, Toxicology, Pharmacology and therapeutics, pain medicine,<br>Hits: 81<br>Dato: 27/02/21<br>Title/abstract screen: 81<br>Full text screen: 0<br>Included: 0<br>**New search 21/03/23**<br>Hits 192<br>Dublicates removed: 79<br>Full text screening: 0<br>Included: 0 |

| clinicaltrial.gov | |
|---|---|
| **Search string** | **Additional information** |
| paclitaxel or docetaxel and Breast Cancer | Filters: Adult (18-65 years), Older adult (>65 years)<br>Hits: 134<br>Dublicate removed: 1<br>Dato: 26/02/21<br>**New search 20/03/23:**<br>Hits: 135<br>Dublicates removed: 108<br>Included: 0 |

# Appendix B

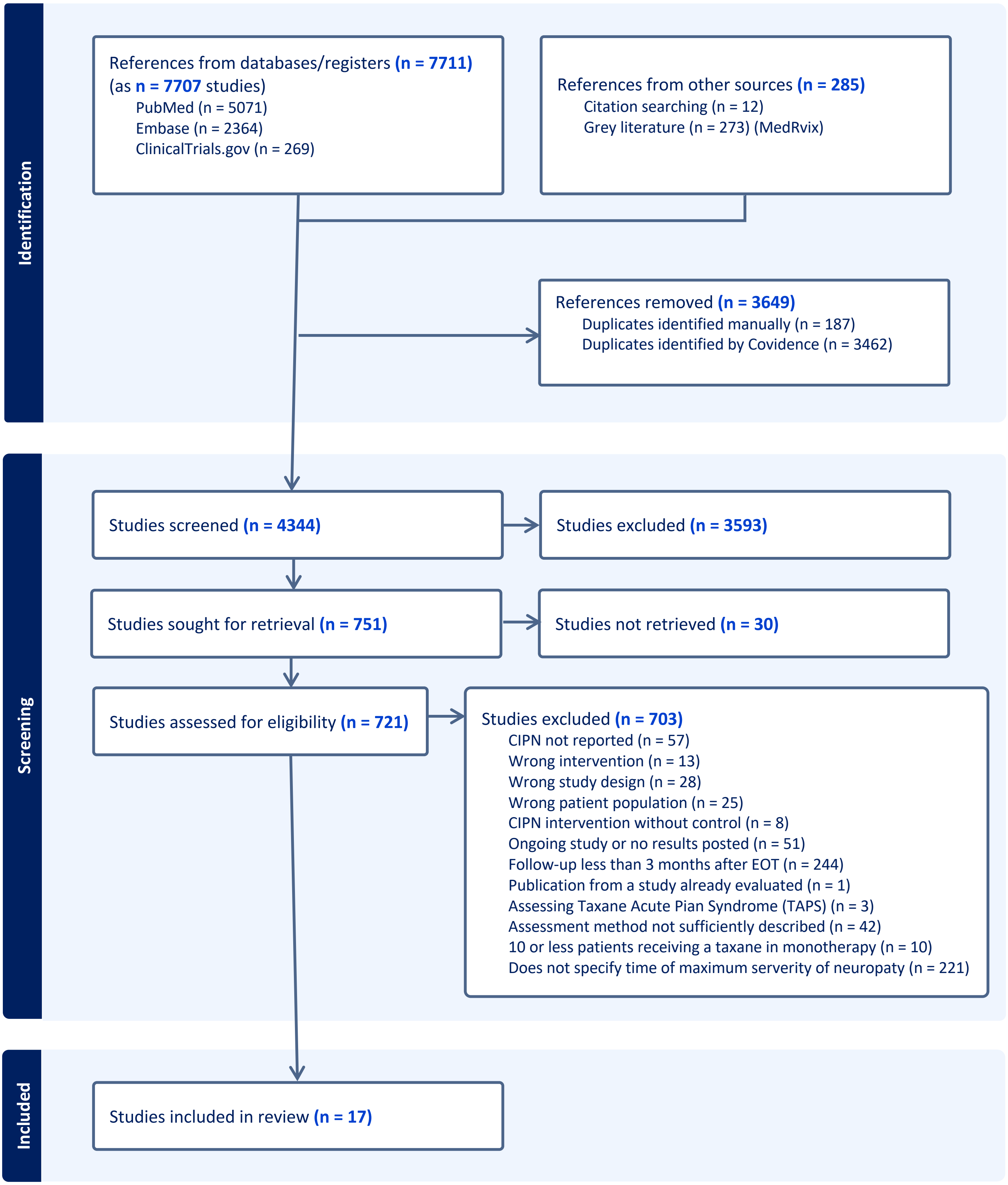
Identification
References from databases/registers (n = 7711)
(as n = 7707 studies)
PubMed (n = 5071)
Embase (n = 2364)
ClinicalTrials.gov (n = 269)
References from other sources (n = 285)
Citation searching (n = 12)
Grey literature (n = 273) (MedRvix)
References removed (n = 3649)
Duplicates identified manually (n = 187)
Duplicates identified by Covidence (n = 3462)
Screening
Studies screened (n = 4344)
Studies excluded (n = 3593)
Studies sought for retrieval (n = 751)
Studies not retrieved (n = 30)
Studies assessed for eligibility (n = 721)
Studies excluded (n = 703)
CIPN not reported (n = 57)
Wrong intervention (n = 13)
Wrong study design (n = 28)
Wrong patient population (n = 25)
CIPN intervention without control (n = 8)
Ongoing study or no results posted (n = 51)
Follow-up less than 3 months after EOT (n = 244)
Publication from a study already evaluated (n = 1)
Assessing Taxane Acute Pian Syndrome (TAPS) (n = 3)
Assessment method not sufficiently described (n = 42)
10 or less patients receiving a taxane in monotherapy (n = 10)
Does not specify time of maximum serverity of neuropaty (n = 221)
Included
Studies included in review (n = 17)

# Appendix C

## Coasting related to Taxane-induced Peripheral Neuropathy in Patients with Breast Cancer: A Systematic Review.

Protocol

*Author(s): Freja L. Kruse[a,b], Margrethe B. Bille[b], Maria E. Lendorf[a,c], Steffen Birk[b]*

*Author affiliations:*

*a: Department of Oncology, Rigshospitalet, Copenhagen, Denmark*

*b: Department of Clinical Neurophysiology, Rigshospitalet, Copenhagen, Denmark*

*c: Department of Oncology and Palliative care, Nordsjællands Hospital, Hillerød, Denmark*

*This protocol was made using the PRISMA-P guidelines(1)*

## Content

Introduction ..... 5
- Rationale ..... 5
- Objective ..... 5

Methods ..... 6
- Eligibility criteria ..... 6
- Information sources ..... 7
- Search Strategy ..... 7
- Study records ..... 8
- Data items ..... 9
- Outcomes and prioritization ..... 9
- Risk of bias in individual studies ..... 9
- Data synthesis ..... 10
- Meta-bias ..... 10
- Confidence in cumulative evidence ..... 10

References ..... 11

# Introduction

## Rationale

Chemotherapy induced peripheral neuropathy (CIPN) is a common side effect for patients with cancer. Affecting 68% withing the first month of the end of therapy overall, the incidence depends on multiple factors such as type of chemotherapy and dosing regimen(2). It is most frequent in patients treated with the following chemotherapeutics: taxanes, platinum compounds, vinca alkaloids, thalidomide, bortezomib and epothilones.(3) The neuropathies present a challenge to the treatment of the patients, as there is currently no recommended strategy for prevention or treatment, aside from a small benefit of duloxetin(4). So, when the neuropathies become too severe, dose-modifications are the only available option. This may in turn affect survival.

Usually the neuropathy presents itself during the treatment period and symptoms improve after discontinuation of chemotherapy, though it may persist for years.(2,5) However it has been observed in some studies, that the CIPN may progress after the end of treatment. A phenomenon called coasting.(3–7)

The coasting phenomenon is often mentioned and observed in clinical studies and reviews. However, to the knowledge of this reviewer it has not been formally investigated as a primary outcome.

Coasting is well known in patients receiving platinum compounds and vinca alkaloids, where it happens in around a third of patients(4,7,8) however it is unclear how often, if at all, it occurs in patients receiving taxanes.(3,5,6,9) Given that that treatment I difficult. The phenomenon poses a problem. There are many interventions aiming to prevent taxane-induced peripheral neuropathy (TIPN), but how they affect the course of the neuropathies is difficult to establish without first establishing the normal occurrence of coasting when using taxanes, to better inform patients and better evaluate interventions towards preventing TIPN.

Studies of CIPN are generally challenged by inconsistency in the kind of assessment methods used to evaluate the patients for symptoms or sign of neuropathy. This have made it difficult to compare studies in the past, however in recent years more and more studies utilizer the same methods(10). It is also important to establish if the method of assessment impact the observation of coasting.

## Objective

The aim of this study is to identify clinical studies that should be able to observe coasting in patients receiving taxanes, defined by sufficient follow-up period and assessment methods. Through analyzing these studies may be possible to assess the occurrence of coasting in TIPN and possibly the severity and challenges when observing the phenomenon.

# Methods

## Eligibility criteria

### *Study design:*

Patients with breast cancer, who have been treated with taxanes in monotherapy. The patients may not have received any of chemotherapies known to frequently course CIPN, as mentioned in *Background*. Other types of chemotherapies or antineoplastic agent may be given.

The studies included must sufficiently describe the assessment method for chemotherapy-induced peripheral neuropathy and may include interventions towards it, but only patients receiving standard treatment or placebo will be included in analysis.

To avoid selection and publication bias. The outcome of interest, in this case coasting, will not be a part of the selection criteria, it only must be possible to observe it. The minimum follow-up is 3 months after the end of treatment (EOT). This limit was chosen to be far enough from end of treatment, which is this study is defined as 3 weeks after the last day of chemotherapy, where progression or debut in neuropathy is to be expected. Still the follow up is short enough, not to exclude to many relevant studies.(2)

The study will include both published and unpublished studies when they meet inclusion and exclusion criteria.

### *PICO definitions in the study:*

P: Patients with breast cancer

I: Taxane treatment in monotherapy

C: Patients developing TIPN, that either diminishes after EOT or is persistent but not progressive.

O: Patients, that develop TIPN, that either develop or progress after EOT, i.e. the coasting phenomenon

### *Inclusion criteria:*

- Clinical studies designed to detect CIPN if present
- Patients included in the study must be >18 years old.
- The patients receive taxane drugs in monotherapy
- Patients with breast cancer
- Longitudinal study design

- Published or unpublished studies

*Exclusion criteria:*

- Case-reports, conference abstracts, expert opinion, reviews or news articles
- Patients included 10 or less
- Any of the following chemotherapeutics given at any point: platinum compounds, vinca alkaloids, thalidomide, bortezomib and epothilones
- The study does not specify or describe the method for observing or examining neuropathies or when during the study period, the assessment was conducted
- The study does not specify when the maximum grade of neuropathy occurred or if none was observed
- Follow-up of toxicities shorter than 3 months after end of treatment
- Other language than English
- Animal or in vitro studies

## Information sources

Systematic searches of the following databases: MEDLINE, EMBASE, Clinicaltrials.gov and medrivx.gov.

These databases where chosen, as the domain of this study is primarily medical, we do not expect to find many added references from searching Web of Science and Google scholar(11) and as resources are scared, this is not done to priorities searching grey literature in clincaltrials.gov and medrivx.gov.

Additionally, hand-searches of relevant articles will be done.

Systematic searches will be done during February 2021.

## Search Strategy

Example of a searches in MEDLINE (via PubMed) and Ovid (Embase):

**PubMed:**

- **Mesh:**
  - **(((( "Taxoids/adverse effects"[Mesh] OR "Taxoids/toxicity"[Mesh] )) AND ("Breast Neoplasms"[Mesh])) NOT ((("Oxaliplatin"[Mesh]) OR "Carboplatin"[Mesh])) NOT ("Platinum Compounds"[Mesh]))**

        - **Filters: English**
        - **Hits: 1501**
- **Title/abstract:**
    - (((((((paclitaxel[Title/Abstract]) OR (taxane*[Title/Abstract])) OR (taxol[Title/Abstract])) OR (docetaxel[Title/Abstract])) OR (taxotere[Title/Abstract])) AND (breast cancer[Title/Abstract])) AND (((((sae) OR (ae)) OR (adverse effect)) OR (toxicity)) OR (tolerability))) NOT (((oxaliplatin[Title/Abstract]) OR (cisplatin[Title/Abstract])) OR (carboplatin[Title/Abstract]) AND (english[Filter]))
        - **Filters: English, last 5 years**
        - **Hits:** 1196

**Embase:**

- Suggestion 1: exp paclitaxel/ae, to [Adverse Drug Reaction, Drug Toxicity] OR exp docetaxel/ae, to [Adverse Drug Reaction, Drug Toxicity] AND exp breast cancer/
    - limit 5 to (english language and exclude medline journals and english)
    - Hits 788
- Suggestion 2:

| | | | |
|---|---|---|---|
| ☐ | 14 | exp paclitaxel/ae, to [Adverse Drug Reaction, Drug Toxicity] | 16604 |
| ☐ | 15 | exp docetaxel/ae, to [Adverse Drug Reaction, Drug Toxicity] | 11482 |
| ☐ | 16 | exp breast cancer/ | 484145 |
| ☐ | 17 | 14 or 15 | 24323 |
| ☐ | 18 | 16 and 17 | 6821 |
| ☐ | 19 | exp breast cancer/ not cisplatin/ not oxaliplatin/ not carboplatin.mp. [mp=title, abstract, heading word, drug trade name, original title, device manufacturer, drug manufacturer, device trade name, keyword, floating subheading word, candidate term word] | 464722 |
| ☐ | 20 | 17 and 19 | 4278 |
| ☐ | 21 | limit 20 to exclude medline journals | 592 |

## Study records

COVIDENCE will be used to manage records during the selection process.

Studies will be assessed by one reviewer screening the title and abstract to assess eligibility. After this the full text will be read. Any studies found to no longer match the study criteria will be excluded.

Included studies will be assessed for heterogeneity in study design to determine which, if any, of them can be included in a meta-analysis.

## Data items

List of data items:

- Trial characteristics (study type, number of patients included number of patients for analysis, primary and secondary outcome).
- Participant characteristics (Age, sex, diagnosis, comorbidities, peripheral neuropathy at baseline).
- Chemotherapy regiment for the patients eligible for this study (both the taxanes and any other given) and additional anti-neoplastic treatment.
- Assessment method for neuropathy.
- Incidence of neuropathy.
- Incidence, course, and grade of coasting.

## Outcomes and prioritization

Primary:

- Incidence of coasting in taxane-induced peripheral neuropathy in patients with breast cancer.

Secondary:

- Severity of neuropathies that develop or progress after end of treatment.

## Risk of bias in individual studies

At study level

- Characteristics of included studies
- Joanne Briggs Critical appraisal tool for Prevalence Studies. (12)

At outcome level

- Unpublished studies will be included when meeting criteria.
- Design and characteristics of the studies will be part of the assessment, when evaluating bias

## Data synthesis

Eligible studies will be assessed to figure out if meta-analysis will be possible. A meta-analysis will be done if available studies are sufficiently homogenous in design and assessment methods.

If meta-analysis is not possible a narrative analysis will be done.

The following methods may be utilized depending on the available studies:

Fixed effect, depending on the results of a $X^2$ test of heterogeneity. If possible, a forest plot will be drawn.

## Meta-bias

- Study quality based on risk of bias evaluations of individual studies.
- Publication bias:
  - The study will include both publishes and unpublished studies.
- Selection bias
  - The studies will be checked for available protocol if possible given resources.
- Funnel plots will be done, if meta-analysis is done.

## Confidence in cumulative evidence

As this is not a guideline, and resources are limited, the GRADE approach will not be utilized.

## References


1. Moher D, Shamseer L, Clarke M, Ghersi D, Liberati A, Petticrew M, et al. Preferred reporting items for systematic review and meta-analysis protocols (PRISMA-P) 2015 statement. Revista Espanola de Nutricion Humana y Dietetica. 2016;20(2).
2. Seretny M, Currie GL, Sena ES, Ramnarine S, Grant R, Macleod MR, et al. Incidence, prevalence, and predictors of chemotherapy-induced peripheral neuropathy: A systematic review and meta-analysis. Vol. 155, Pain. 2014.
3. Grisold W, Cavaletti G, Windebank AJ. Peripheral neuropathies from chemotherapeutics and targeted agents: Diagnosis, treatment, and prevention. Neuro Oncol. 2012;14(SUPPL.4).
4. Loprinzi CL, Lacchetti C, Bleeker J, Cavaletti G, Chauhan C, Hertz DL, et al. Prevention and management of chemotherapy-induced peripheral neuropathy in survivors of adult cancers: ASCO guideline update. Journal of Clinical Oncology. 2020;38(28).
5. Eckhoff L, Knoop A, Jensen M, Ewertz M. Persistence of docetaxel-induced neuropathy and impact on quality of life among breast cancer survivors. Eur J Cancer. 2015;51(3).
6. Kerckhove N, Collin A, Condé S, Chaleteix C, Pezet D, Balayssac D. Long-term effects, pathophysiological mechanisms, and risk factors of chemotherapy-induced peripheral neuropathies: A comprehensive literature review. Vol. 8, Frontiers in Pharmacology. 2017.
7. Argyriou AA, Bruna J, Marmiroli P, Cavaletti G. Chemotherapy-induced peripheral neurotoxicity (CIPN): An update. Vol. 82, Critical Reviews in Oncology/Hematology. 2012.
8. Verstappen CCP, Koeppen S, Heimans JJ, Huijgens PC, Scheulen ME, Strumberg D, et al. Dose-related vincristine-induced peripheral neuropathy with unexpected off-therapy worsening. Neurology. 2005;64(6).
9. Duggett NA, Griffiths LA, McKenna OE, de Santis V, Yongsanguanchai N, Mokori EB, et al. Oxidative stress in the development, maintenance and resolution of paclitaxel-induced painful neuropathy. Neuroscience. 2016;333.
10. Molassiotis A, Cheng HL, Lopez V, Au JSK, Chan A, Bandla A, et al. Are we mis-estimating chemotherapy-induced peripheral neuropathy? Analysis of assessment methodologies from a prospective, multinational, longitudinal cohort study of patients receiving neurotoxic chemotherapy. BMC Cancer. 2019;19(1).
11. Bramer WM, Rethlefsen ML, Kleijnen J, Franco OH. Optimal database combinations for literature searches in systematic reviews: A prospective exploratory study. Syst Rev. 2017;6(1).
12. Munn Z, MClinSc SM, Lisy K, Riitano D, Tufanaru C. Methodological guidance for systematic reviews of observational epidemiological studies reporting prevalence and cumulative incidence data. Int J Evid Based Healthc. 2015;13(3).

## Appendix D - Protocol Amendments Log:

Method for decisions and registration of amendments:

- Any amendments to the protocol must be discusses among the authors to achieve consensus before implementation.
- The study protocol, which is available in Appendix C, is the updated version with all amendments added. Any chances and reasons for changes since the protocol was first finished, can be found below
- Minor changes to wording are not registered

**Date of finish protocol: 23/2/21**

### Inclusion and exclusion criteria:

Original at date of finish protocol:

*Inclusion criteria:*

- Clinical studies that include attempt to observe CIPN
- Patients included in the study must be >18 years old.
- The patients receive taxane drugs in monotherapy
- Patients with breast cancer
- Longitudinal setup
- Published or unpublished studies

*Exclusion criteria:*

- Phase 1 studies, case-reports, conference abstracts
- Patients included less than 10
- Any of the following chemotherapeutics given platinum compounds, vinca alkaloids, thalidomide, bortezomib and epothilones
- The study does not specify or describe the method for observing or examining neuropathies or when during the study period, the assessment was conducted
- Other language than English
- Animal studies

## Date of amendment: 27/3/21:

**Amended version:**

*Inclusion criteria:*

- Clinical studies that include attempt to observe CIPN
- Patients included in the study must be >18 years old.
- The patients receive taxane drugs in monotherapy
- Patients with breast cancer
- Longitudinal setup
- Published or unpublished studies

*Exclusion criteria:*

- Case-reports, conference abstracts, expert opinion, reviews or news articles
- Patients included 10 or less
- Any of the following chemotherapeutics given at any point: platinum compounds, vinca alkaloids, thalidomide, bortezomib and epothilones
- The study does not specify or describe the method for observing or examining neuropathies or when during the study period, the assessment was conducted
- The study does not specify when the maximum grade of neuropathy occurred or if none was observed
- Follow-up shorter than 3 months after end of treatment
- Other language than English
- Animal or in vitro studies

**Reason for amendment:**

- Scarcity of resources demanded stricter criteria.
- The follow up period restriction and specification of type of studies and number of patients was originally intended but by mistake not written
- The exclusion of studies not specifying the time of maximum neuropathy grade was added after the full text assessment began. It became clear the studies not specifying this could not be used to assess incidence of coasting if the course of the neuropathy is not clarified.

## Date of amendment: 13/4/21:

The definition of taxane in monotherapy was amended to not include patients receiving anti-HER2 treatment concurrently.

**Reason for amendment:** This was done to bring down the number of included studies and to avoid possible confounding. Studies that would be possible for inclusion where marked, to be easy to re-visit if later analysis was possible

## Date of amendment: 22/4/21:

The definition of taxane in monotherapy was amended to again include patients receiving anti-HER2 treatment concurrently.

**Reason for amendment:** This was done as there were too few studies of prober quality and size left, and reporting of concurrent anti-HER2 therapy was not always reported, though due to the high impact anti-HER2 therapy have had on breast cancer treatment in the early 2000[1,2], it must be assumed that patients, that are HER2-postive would receive anti-HER2 treatment. Though it may be a confounding factor, it was considered necessary to include. There is no available literature to support, that it causes or exacerbates CIPN[3]

**References:**


1. Korde LA, Somerfield MR, Carey LA, et al. Neoadjuvant Chemotherapy, Endocrine Therapy, and Targeted Therapy for Breast Cancer: ASCO Guideline. *J Clin Oncol.* 2021;39(13):1485-1505.
2. Romond EH, Perez EA, Bryant J, et al. Trastuzumab plus adjuvant chemotherapy for operable HER2-positive breast cancer. *N Engl J Med.* 2005;353(16):1673-1684.
3. Sodergren SC, Copson E, White A, et al. Systematic Review of the Side Effects Associated With Anti-HER2-Targeted Therapies Used in the Treatment of Breast Cancer, on Behalf of the EORTC Quality of Life Group. *Target Oncol.* 2016;11(3):277-292.


## Date of amendment: 12/12/21:

**Amendment:** Tool for quality assessment for included studies is changed from ROBINS-I and Rob2 til Joanna Briggs Checklist for Prevalence Studies

**Reason for amendment:** Joanna Briggs Checklist for Prevalence Studies are better at assessing the studies quality in terms of finding the prevalence of coasting.

# Supplementary F: Critical appraisal

## Statistical calculation of sample size

Using the calculation recommended by JBI (Naing et al 2006)

n= sample size

Z = Z statistic for a level of confidence

P = Expected prevalence or proportion (in proportion of one; if 10%, P = 0.1), 10% was chosen based om (eckhoff et al 2015)

d = precision (in proportion of one; if 5%, d=0.05)

$$n = \frac{Z^2 * P * (1 - P)}{d^2}$$

$$n = \frac{1{,}96^2 * 0{,}1 * (1 - 0{,}1)}{0{,}05^2} = 138{,}29$$

The sample size needed for 80% power to detect coasting if prevalent in 10% of patients is 138.

## Short overview of the critical appraisal system as applied for this review

| | Yes | No | Unclear | Not applicable |
|---|---|---|---|---|
| 1. Was the sample frame appropriate to address the target population? | Characteristics, demographic and medical history of the population. | | | |
| 2. Were study participants sampled in an appropriate way? | Sampling. Approched at clinic, participant in another cohort or study. Convinience sample. | | | |
| 3. Was the sample size adequate? | >138 | <138 | | |
| 4. Were the study subjects and the setting described in detail? | Sufficient description of participants. | | | |
| 5. Was the data analysis conducted with sufficient coverage of the identified sample? | Any specific characteristics in non repsonders compared to responders. | | | |
| 6. Were valid methods used for the identification of the condition? | PRO's EORTC-CIPN20, FACT-ntx and PNQ are validated, so is CTCAE but have problems with interrater realiability and inconsistensies in patients compared to health care workers. TNS is also validated. A combination of modalities is preferred. | | | |
| 7. Was the condition measured in a standard, reliable way for all participants? | Who and how was assesments done. Was assessers trained? | | | |
| 8. Was there appropriate statistical analysis? | This is difficult to apply to coasting, as it is an individual event. As such it is not expected that statistical | | | |

| | |
|---|---|
| | analysies is applied for this, unless individual symptom developments is a endpoint. |
| 9. Was the response rate adequate, and if not, was the low response rate managed appropriately? | Characteristics of patients lost to follow-up and the amount of patients lost to follow up. More than 20% is considered significant. |

## JBI CRITICAL APPRAISAL CHECKLIST FOR STUDIES REPORTING PREVALENCE DATA

Reviewer: Freja Løvendal Kruse, Date: 08/01/22

Author: Bandos, Year: 2018, Record Number: 1

| | Yes | No | Unclear | Not applicable |
|---|---|---|---|---|
| 10. Was the sample frame appropriate to address the target population? | X | □ | □ | □ |
| 11. Were study participants sampled in an appropriate way? | x[a] | □ | □ | □ |
| 12. Was the sample size adequate? | X | □ | □ | □ |
| 13. Were the study subjects and the setting described in detail? | x[b] | □ | □ | □ |
| 14. Was the data analysis conducted with sufficient coverage of the identified sample? | X[a+b] | □ | □ | □ |
| 15. Were valid methods used for the identification of the condition? | □ | x[c] | □ | □ |
| 16. Was the condition measured in a standard, reliable way for all participants? | □ | x[d] | □ | □ |
| 17. Was there appropriate statistical analysis? | x | □ | □ | □ |
| 18. Was the response rate adequate, and if not, was the low response rate managed appropriately? | □ | □ | x[e] | □ |

Overall appraisal: Include x Exclude □ Seek further info □

Comments (Including reason for exclusion)

a) In Ganz 2011 (13), patients not participating in the QOL substudy had primarily already started the treatment or were inrolled in B30 after closure of the substudy. Though there were some factors to take into consideration:

> Women who participated in the QOL substudy and were included in the current analyses were somewhat younger (<50 years; 50.1% vs 42.2%) and less obese (37.5% vs 32.4% with normal body mass index [BMI]) than the rest of the patients in the trial. Slightly more white women had data that were included in these analyses (86.2% vs 81.0%).

a.

b) From Ganz 2011: *"MH and QOL questionnaires were administered at baseline (after consent, but before first treatment), and at office visits at the time of cycle 4, day 1 (during chemotherapy), and then at 6, 12, 18, and 24 months. Questionnaires were completed at an in-person visit, with mail and telephone collection as alternatives. A missing data form was completed if the woman declined to complete a scheduled questionnaire or for an administrative error in data collection. Compliance monitoring occurred through clinical site–specific updates on missing data, tailored letters, and telephone contacts from the*

*NSABP Biostatistical Center. Patients received individualized calendars (in a monthly format, covering the entire time frame for assessments) to record menstrual cycles as an aid for completing the MH questionnaire. (Calendars were not submitted.)".* Aside from this, information on comorbitities was not shown. Peripheral neuropathy (PN) at baseline was not mentioned.

c) The study use the Breast Cancer Prevention Trail Symptom Checklist (BCPT), which is a non validated metod for assessing Chemotherapy Induced Peripheral Neuropathy (CIPN), They specifically ask about tingling/numbness and how much it "bothered" the patient at each assessment.
d) g) The study design is good for their primary outcome, but this was not CIPN and the assessment method is not validated.
e) The method is not validated, but the way it was administered was standardizedfor all patients, an missing information handled with discretion of the group. It does not say if it was several people administering the questionaries and how they were trained.
f) It doesn't say if there were any dropouts during follow up or how they were handled.

## JBI CRITICAL APPRAISAL CHECKLIST FOR STUDIES REPORTING PREVALENCE DATA

Reviewer: Freja Løvendal Kruse, Date: 08/01/22

Author: Heather Greenlee, Year: 2017, Record Number: 2

| | Yes | No | Unclear | Not applicable |
|---|---|---|---|---|
| 1. Was the sample frame appropriate to address the target population? | X[a] | □ | □ | □ |
| 2. Were study participants sampled in an appropriate way? | X[b] | □ | □ | □ |
| 3. Was the sample size adequate? | X | □ | □ | □ |
| 4. Were the study subjects and the setting described in detail? | x[b,c] | □ | □ | □ |
| 5. Was the data analysis conducted with sufficient coverage of the identified sample? | x[f] | □ | □ | □ |
| 6. Were valid methods used for the identification of the condition? | x[d] | □ | □ | □ |
| 7. Was the condition measured in a standard, reliable way for all participants? | x[e] | □ | □ | □ |
| 8. Was there appropriate statistical analysis? | X | □ | □ | □ |
| 9. Was the response rate adequate, and if not, was the low response rate managed appropriately? | □ | x[f] | □ | □ |

Overall appraisal: Include x Exclude □ Seek further info □

Comments (Including reason for exclusion):

a) The population was from the KPNC register.

b) It's was well described in the text or in Kwan 2008

c) Need for more information about exposure: In this study, the exposure is taxan treatment. There is no information about how this information was collected, also information about regime, accumulated dosis and resons for early cession of treatment.

d) FACT-Ntx is validated. Follow up was at 6 and 24 months. Coasting could have happened within this time.

e) From Kwan 2008: *For quality control purposes, yearly re certifications are conducted among all interviewers in which the field staff coordinator reviews the baseline interview protocols and procedures with the interviewer at the study coordinating center and then observes a live baseline interview in the field. Appropriate feedback and corrective action is taken as necessary to ensure standardization of the interview process among all field staff. Furthermore, quality assurance*

*procedures, including review of baseline files by two separate staff members, are conducted at the study coordinating center to provide an additional check of data collection consistency. Participants are called back for clarification if necessary.*

---

f) After 6 months the responsrate was 37% and at 24 months 56%. It was not described how lost to follow up was handled., but certain characteristics for no-responders was mentioned: *A total of 771 (62.3%) women completed the FACT-NTX questionnaire at six months, and 544 (44.8%) at 24 months. Compared with those who provided data at six and 24 months, women who did not provide data were more likely to be younger, to be African American, to have lower education attainment, to be obese, to eat fewer fruit/vegetables, to spend less time in MVPA, and to be nonusers of antioxidants*

## JBI CRITICAL APPRAISAL CHECKLIST FOR STUDIES REPORTING PREVALENCE DATA

Reviewer: Freja Løvendal Kruse, Date: 02/02/22

Author: Dawn Hershman, Year: 2011 , Record Number: 3

| | Yes | No | Unclear | Not applicable |
|---|---|---|---|---|
| 1. Was the sample frame appropriate to address the target population? | x[a] | □ | □ | □ |
| 2. Were study participants sampled in an appropriate way? | □ | □ | x[b] | □ |
| 3. Was the sample size adequate? | □ | X | □ | □ |
| 4. Were the study subjects and the setting described in detail? | □ | X[c] | □ | □ |
| 5. Was the data analysis conducted with sufficient coverage of the identified sample? | □ | X[d] | □ | □ |
| 6. Were valid methods used for the identification of the condition? | x[e] | □ | □ | □ |
| 7. Was the condition measured in a standard, reliable way for all participants? | x | □ | □ | □ |
| 8. Was there appropriate statistical analysis? | □ | □ | x[f] | □ |
| 9. Was the response rate adequate, and if not, was the low response rate managed appropriately? | □ | □ | x | □ |

Overall appraisal: Include x Exclude □ Seek further info □

Comments (Including reason for exclusion)

a) Sample information is sex, tumorstage I-III and about to initiate adj. paclitaxel therapy.

b) The cross sectional part (not the one analysed for this review) is described as chosen at rutine follow up visits at the oncology clinic. But it doesn't say how the cohort was chosen.

c) The following was described in methods:

"*Demographic and medical information were collected using self-report measures and via medical chart review. Selfreport data included age, race, ethnicity, marital status, educational level, current employment status, smoking history, and alcohol consumption. Medical data were abstracted from the most recent chart notes and included height, weight, body mass index (BMI), menopausal status, date of breast cancer diagnosis, stage at diagnosis, tumor hormone receptor and HER2/neu status, paclitaxel regimen, dates of paclitaxel treatments, highest grade of neuropathy during treatment (according to NCI Common Terminology Criteria for Adverse Events, Version 3.0) [10], paclitaxel dose delays or reductions, other adjuvant chemotherapy, radiation and hormonal therapy, and chronic illnesses that may predispose to peripheral neuropathy (such as diabetes).*"

But results, such as comorbidities, PN before baseline, smoking, employment and marital status and other treatment was not described in results or tables.

d) There is no description of any drop out over time in results. It was not described how the screening was done. Only that they were unaware of the study hypothesis and the very few decline to participate and those that did primarily was dure to timeconstraints.

e) The methods were CTCAE v v3.0, FACT-Ntx, VT (Vibration threshold, biothesiometer) and TT (tactile threshold - von Freys filaments). The FACT-Ntx is a validated PRO and there was both subjective and objective measures.

f) In the analysis if the association between chance over time in FACT-Ntx scores from baseline and QST scores, the controlled for age. Though one could argue that age is a moderator and not a confounder. Since age may increase the risk of worse neuropathy and as such the scores?

## JBI CRITICAL APPRAISAL CHECKLIST FOR STUDIES REPORTING PREVALENCE DATA

Reviewer: Freja Løvendal Kruse, Date: 23/12/21

Author: Dawn Hershman, Year: 2018 Record Number: 4

| | Yes | No | Unclear | Not applicable |
|---|---|---|---|---|
| 10. Was the sample frame appropriate to address the target population? | X | □ | □ | □ |
| 11. Were study participants sampled in an appropriate way? | □ | □ | x[a] | □ |
| 12. Was the sample size adequate? | X | □ | □ | □ |
| 13. Were the study subjects and the setting described in detail? | x | □ | □ | □ |
| 14. Was the data analysis conducted with sufficient coverage of the identified sample? | X | □ | □ | □ |
| 15. Were valid methods used for the identification of the condition? | x[b] | □ | □ | □ |
| 16. Was the condition measured in a standard, reliable way for all participants? | x[b] | □ | □ | □ |
| 17. Was there appropriate statistical analysis? | X[d] | □ | □ | □ |
| 18. Was the response rate adequate, and if not, was the low response rate managed appropriately? | x[c] | □ | □ | □ |

Overall appraisal: Include x Exclude □ Seek further info □

Comments (Including reason for exclusion)

a) It doesn't say how the participants were found.

b) FACT-Ntx at baseline, week 12, 24, 36, 52 and 104

*c)* From the text: *The number of patients with available FACT-NTX scores diminished over time, but was similar between arms. Missing outcome data at each time point did not exceed 5% in either treatment group and did not differ over time by group. The number of evaluable patients in the study also did not differ statistically significantly over time by arm.*

*d)* *"Baseline demographic factors were compared between treatment groups using chi-square tests for categorical variables and t tests for continuous variables (all two-sided tests). We examined the effect of ALC on CIPN, as measured by FACT-NTX score, over two years using linear mixed models for longitudinal data to account for correlated outcomes within patients over time. The outcome is NTX score at each time point except baseline. We specified a random intercept and slope for time and adjusted for the protocol specified stratification factors (planned taxane-based chemotherapy and age colony-stimulating*

*factor). Predictive factors were identified by testing the interaction of each baseline factor with treatment arm. If no interaction was evident (P > .05), then data were combined across arms, and the potential prognostic effects of the different factors were examined, adjusting for treatment arm as a covariate. Logistic regression was used to explore associations between these predictive factors and risk of a five-point decrease in FACT-NTX at year 1 and year 2. The study was designed to detect a minimally important difference of three points in the FACT-NTX score (10). We prespecified a test of the intervention effect on the FACT-NTX over the full two years of assessments as the single primary examination, at a two-sided alpha of .05. All other tests were considered exploratory, with two-sided P values provided. All models were fit in R version 3.3.1 (R Core Team, 2013) (11) and SAS version 9.4 (SAS Institute, Inc., Cary, NC).”*

## JBI CRITICAL APPRAISAL CHECKLIST FOR STUDIES REPORTING PREVALENCE DATA

Reviewer: Freja Løvendal Kruse, Date: 12/01/22

Author: Kwang-Min Lee, Year: 2018, Record Number: 5

| | Yes | No | Unclear | Not applicable |
|---|---|---|---|---|
| 19. Was the sample frame appropriate to address the target population? | □ | □ | x[a] | □ |
| 20. Were study participants sampled in an appropriate way? | x[b] | □ | □ | □ |
| 21. Was the sample size adequate? | x | □ | □ | □ |
| 22. Were the study subjects and the setting described in detail? | x | □ | □ | □ |
| 23. Was the data analysis conducted with sufficient coverage of the identified sample? | □ | x[d] | □ | □ |
| 24. Were valid methods used for the identification of the condition? | □ | x[c] | □ | □ |
| 25. Was the condition measured in a standard, reliable way for all participants? | □ | □ | X[f] | □ |
| 26. Was there appropriate statistical analysis? | x | □ | □ | □ |
| 27. Was the response rate adequate, and if not, was the low response rate managed appropriately? | □ | □ | x[e] | □ |

Overall appraisal: Include x Exclude □ Seek further info □

Comments (Including reason for exclusion)

a) They excluded patients >70 years, with shift work and psychiatric diagnosis. This was nessesary in this papir, as they were examining the the effects of pre-treatment anxiety for the development of persistent CIPN. But it does make the population quite narrow. From Lee 2017 (the primary study): *Individuals with a history of another cancer or with another significant medical condition, including neurological, endocrinological or ophthalmological disease, were excluded. Individuals with a history of psychiatric treatment for more than 1 month before chemotherapy were ineligible, because that could affect the mood and sleep of the participants. Those who had shift work in the preceding 6 months were also ineligible, because that could result in short-term circadian disruption.*

*b)* From Lee 2017 (the primary study): *Participants were recruited for this prospective observational study in a tertiary general hospital in Seoul, Republic of Korea from November 2013 to March 2016. Clinical staff approached potential patients who visited an oncologist's office for neoadjuvant chemotherapy and provided detailed information about the study.*

c) The methods for assessing CIPN symptoms is not validated. From the article: *Participants reported their neuropathic symptoms of numbness or tingling during the previous 24 h using a 0–10 numerical rating scale. A score of 0 indicated no symptoms, while 10 indicated severe symptoms that were intolerable. Consistent with other studies of cancer-related pain and numbness, we found that moderate neuropathy symptoms were indicated at a grade of 3 or higher on the numerical scale [17,19]. Persistent CIPN was defined as numbness or tingling sensation occurring in the last chemotherapy cycle and persisting over 8 months after completion of chemotherapy.* It also seems problematic that only symptoms in the last 24 hours was considered.

d) The mean age was 44,17 years SD 7,25 and only 25% >50 years. So the population was quite young. Patient >70 years was excluded.

---

e) 17,5% dropped out. Of the 23 people was lost to follow up, 2 changed treatment plan and 7 had another chemotherapeutic regiment. It did not say if there were different characteristics in this group.

---

f) It did not state how the CIPN questions were presented or answered.

## JBI CRITICAL APPRAISAL CHECKLIST FOR STUDIES REPORTING PREVALENCE DATA

Reviewer: Freja Løvendal Kruse, Date: 12/01/22

Author: Miguel Martin, Year: 2008, Record Number: 6

| | Yes | No | Unclear | Not applicable |
|---|---|---|---|---|
| 28. Was the sample frame appropriate to address the target population? | X | □ | □ | □ |
| 29. Were study participants sampled in an appropriate way? | □ | □ | X | □ |
| 30. Was the sample size adequate? | x | □ | □ | □ |
| 31. Were the study subjects and the setting described in detail? | □ | □ | x | □ |
| 32. Was the data analysis conducted with sufficient coverage of the identified sample? | x | □ | □ | □ |
| 33. Were valid methods used for the identification of the condition? | X[a] | □ | □ | □ |
| 34. Was the condition measured in a standard, reliable way for all participants? | □ | □ | X | □ |
| 35. Was there appropriate statistical analysis? | □ | □ | X | □ |
| 36. Was the response rate adequate, and if not, was the low response rate managed appropriately? | □ | □ | X | □ |

Overall appraisal: Include x Exclude □ Seek further info □

Comments (Including reason for exclusion)

a) CTCAE is a well recognised tool. But has problems with interrater reliability

## JBI CRITICAL APPRAISAL CHECKLIST FOR STUDIES REPORTING PREVALENCE DATA

Reviewer: Freja Løvendal Kruse, Date 13/01/22

Author: Ding Quan Ng, Year: 2020, Record Number: 7

| | Yes | No | Unclear | Not applicable |
|---|---|---|---|---|
| 37. Was the sample frame appropriate to address the target population? | □ | X[b] | □ | □ |
| 38. Were study participants sampled in an appropriate way? | □ | □ | x[e] | □ |
| 39. Was the sample size adequate? | □ | x | □ | □ |
| 40. Were the study subjects and the setting described in detail? | x | □ | □ | □ |
| 41. Was the data analysis conducted with sufficient coverage of the identified sample? | □ | □ | x[d] | □ |
| 42. Were valid methods used for the identification of the condition? | x[a] | □ | □ | □ |
| 43. Was the condition measured in a standard, reliable way for all participants? | □ | □ | x[c] | □ |
| 44. Was there appropriate statistical analysis? | X | □ | □ | □ |
| 45. Was the response rate adequate, and if not, was the low response rate managed appropriately? | □ | □ | x[d] | □ |

Overall appraisal: Include x Exclude □ Seek further info □

Comments (Including reason for exclusion):

a) Good assesment strategy. PNQ, EORTC-CIPN20 at baseline, EOT, 3, 6 and 9 m. og NCS and SSR at baseline, EOT and 6 m.

b) The patients had to have at central venous access, this may have excluded some patients, finding this too invasive. Also the patient were only of asian ethnicity save 1.

c) The methods is well described, but it doesn't say who performed NCS and SSR or how the patient received PRO's.

d) 5 drop puts from NCS was not explained. This is few, but as it leves only 12 to be analyzed it is a substantial amount. In the PRO's there were no drop puts after baseline, and the ones before were explained in terms of reasons, but not characteristics.

e) It doesn't say how and were participant were chosen/approached.

## JBI CRITICAL APPRAISAL CHECKLIST FOR STUDIES REPORTING PREVALENCE DATA

Reviewer: Freja Løvendal Kruse, Date: 13/01/22

Author: U. Nitz, Year: 2014, Record Number: 8

| | Yes | No | Unclear | Not applicable |
|---|---|---|---|---|
| 46. Was the sample frame appropriate to address the target population? | x[a] | □ | □ | □ |
| 47. Were study participants sampled in an appropriate way? | x | □ | □ | □ |
| 48. Was the sample size adequate? | x | □ | □ | □ |
| 49. Were the study subjects and the setting described in detail? | x[b] | □ | □ | □ |
| 50. Was the data analysis conducted with sufficient coverage of the identified sample? | □ | □ | x[d] | □ |
| 51. Were valid methods used for the identification of the condition? | x[c] | □ | □ | □ |
| 52. Was the condition measured in a standard, reliable way for all participants? | □ | □ | X[b] | □ |
| 53. Was there appropriate statistical analysis? | □ | □ | x[e] | □ |
| 54. Was the response rate adequate, and if not, was the low response rate managed appropriately? | □ | X[d] | □ | □ |

Overall appraisal: Include x Exclude □ Seek further info □

Comments (Including reason for exclusion):

a) Age <65 years were excluded.

b) In terms of neuropathy, it doesn't say who did the evaluations or how they were trained. It does say when.

c) CTCAE v 2.0. See prior description of the pros and cons of this method.

d) 92 was lost to follow up out of 791. It doesn't say why and doesn't specify if there were differences between the groups.

e) Doesn't discripe any use of statistics that look for individual toxicity profiles.

## JBI CRITICAL APPRAISAL CHECKLIST FOR STUDIES REPORTING PREVALENCE DATA

Reviewer : Freja Løvendal Kruse, Date: 13/01/22

Author: Lucile Pabst, Year: 2020 Record Number: 9

| | Yes | No | Unclear | Not applicable |
|---|---|---|---|---|
| 55. Was the sample frame appropriate to address the target population? | □ | x[a] | □ | □ |
| 56. Were study participants sampled in an appropriate way? | x | □ | □ | □ |
| 57. Was the sample size adequate? | x | □ | □ | □ |
| 58. Were the study subjects and the setting described in detail? | x | □ | □ | □ |
| 59. Was the data analysis conducted with sufficient coverage of the identified sample? | x[d] | □ | □ | □ |
| 60. Were valid methods used for the identification of the condition? | x[b] | □ | □ | □ |
| 61. Was the condition measured in a standard, reliable way for all participants? | □ | □ | x[c] | □ |
| 62. Was there appropriate statistical analysis? | X | □ | □ | □ |
| 63. Was the response rate adequate, and if not, was the low response rate managed appropriately? | x[d] | □ | □ | □ |

Overall appraisal: Include x Exclude □ Seek further info □

Comments (Including reason for exclusion):

a) Patients were all aged >65 years. Male patients and those that received incomplete therapy excluded.

b) CTCAE at end of treatment and after 2 years. No baseline measures.

c) CTCAE is critizied for interrater reliability, which may be bettered by training. It does not say who evalueated the patients symptoms or how they were trained.

d) Only 2 was not followed up as they died before the 2 year follow up time. There were no patients lost to follow up reported. This might be a little unlikely, but it is not reported how follow up measurements were conducted. It is not reported is there were specific characteristics for participants, that decline to participate.

## JBI CRITICAL APPRAISAL CHECKLIST FOR STUDIES REPORTING PREVALENCE DATA

Reviewer : Freja Løvendal Kruse, Date 13/01/22

Author: Pace, Year: 2007, Record Number: 10

| | Yes | No | Unclear | Not applicable |
|---|---|---|---|---|
| 64. Was the sample frame appropriate to address the target population? | x | □ | □ | □ |
| 65. Were study participants sampled in an appropriate way? | x | □ | □ | □ |
| 66. Was the sample size adequate? | □ | x | □ | □ |
| 67. Were the study subjects and the setting described in detail? | x | □ | □ | □ |
| 68. Was the data analysis conducted with sufficient coverage of the identified sample? | x | □ | □ | □ |
| 69. Were valid methods used for the identification of the condition? | x[a] | □ | □ | □ |
| 70. Was the condition measured in a standard, reliable way for all participants? | □ | x[b] | □ | □ |
| 71. Was there appropriate statistical analysis? | x | □ | □ | □ |
| 72. Was the response rate adequate, and if not, was the low response rate managed appropriately? | x | □ | □ | □ |

Overall appraisal: Include x Exclude □ Seek further info □

Comments (Including reason for exclusion):

a) TNS, done at baseline, midway (12 weeks) and EOT (24 weeks), for 11 patients at 6 months. There was a wide range in the follow up timing of assessment. Mean 6 months (range 4-17 months).

## JBI CRITICAL APPRAISAL CHECKLIST FOR STUDIES REPORTING PREVALENCE DATA

Reviewer: Freja Løvendal Kruse, Date: 13/01/22

Author: Deirdre Pachman, Year: 2017, Record Number: 11

| | Yes | No | Unclear | Not applicable |
|---|---|---|---|---|
| 73. Was the sample frame appropriate to address the target population? | X | □ | □ | □ |
| 74. Were study participants sampled in an appropriate way? | X | □ | □ | □ |
| 75. Was the sample size adequate? | □ | X | □ | □ |
| 76. Were the study subjects and the setting described in detail? | x | □ | □ | □ |
| 77. Was the data analysis conducted with sufficient coverage of the identified sample? | x | □ | □ | □ |
| 78. Were valid methods used for the identification of the condition? | x[b] | □ | □ | □ |
| 79. Was the condition measured in a standard, reliable way for all participants? | x | □ | □ | □ |
| 80. Was there appropriate statistical analysis? | □ | □ | x[a] | □ |
| 81. Was the response rate adequate, and if not, was the low response rate managed appropriately? | x | □ | □ | □ |

Overall appraisal: Include x Exclude □ Seek further info □

Comments (Including reason for exclusion):

a) It is unclear if statistics that identified individual development of symptoms were used.

b) EORTC-CIPN20 is a validated PRO measure.

## JBI CRITICAL APPRAISAL CHECKLIST FOR STUDIES REPORTING PREVALENCE DATA

Reviewer Freja Lørvendal Kruse Date 17/09/2022

Author Ruddy, Year 2019, Record Number: 12

| | Yes | No | Unclear | Not applicable |
|---|---|---|---|---|
| 82. Was the sample frame appropriate to address the target population? | x[a] | □ | □ | □ |
| 83. Were study participants sampled in an appropriate way? | □ | □ | X[b] | □ |
| 84. Was the sample size adequate? | □ | x | □ | □ |
| 85. Were the study subjects and the setting described in detail? | x | □ | □ | □ |
| 86. Was the data analysis conducted with sufficient coverage of the identified sample? | □ | □ | X[c] | □ |
| 87. Were valid methods used for the identification of the condition? | X | □ | □ | □ |
| 88. Was the condition measured in a standard, reliable way for all participants? | X[d] | □ | □ | □ |
| 89. Was there appropriate statistical analysis? | □ | □ | □ | x |
| 90. Was the response rate adequate, and if not, was the low response rate managed appropriately? | X | □ | □ | □ |

Overall appraisal: Include x Exclude □ Seek further info □

Comments (Including reason for exclusion):

a) Only ethnicity caucasian or 2 unclear on ethnicity. Age appropriate. The aurthors describe that the patients experienced less neuropathy compared to other control groups.

b) Doesn't describe how patients were chosen.

c) Only 3 non-responders. Doesn't specific characteristics for this group.

d) Doesn't specify who perfomed CTCAE assessments. But there were nurse calls to ask how patients were doing and remind about questionaires.

## JBI CRITICAL APPRAISAL CHECKLIST FOR STUDIES REPORTING PREVALENCE DATA

Reviewer: Freja Løvendal Kruse, Date 13/01/22

Author: Kojiro Shimozuma, Year: 2012, Record Number: 13

| | Yes | No | Unclear | Not applicable |
|---|---|---|---|---|
| 91. Was the sample frame appropriate to address the target population? | X | □ | □ | □ |
| 92. Were study participants sampled in an appropriate way? | X | □ | □ | □ |
| 93. Was the sample size adequate? | X | □ | □ | □ |
| 94. Were the study subjects and the setting described in detail? | x[b] | □ | □ | □ |
| 95. Was the data analysis conducted with sufficient coverage of the identified sample? | x | □ | □ | □ |
| 96. Were valid methods used for the identification of the condition? | x[a] | □ | □ | □ |
| 97. Was the condition measured in a standard, reliable way for all participants? | x[b] | □ | □ | □ |
| 98. Was there appropriate statistical analysis? | x | □ | □ | □ |
| 99. Was the response rate adequate, and if not, was the low response rate managed appropriately? | x[c] | □ | □ | □ |

Overall appraisal: Include x Exclude □ Seek further info □

Comments (Including reason for exclusion):

a) PNQ, FACT-Ntx at baseline, before treatment cycles 3, 5, 7 and at 7 months and 1 year after start of adj. therapy.

*b)* From the article: "*Patients were asked to complete the PNQ and FACT questionnaires at the following time points: pretreatment (baseline), before treatment cycles 3, 5, and 7, at 7 months, and at 1 year after starting adjuvant chemotherapy (Fig. 1). The PNQ and FACT questionnaires were distributed to the patients by clinical research coordinators and physicians. Patients completed these questionnaires independently and return them directly to the study data center without getting any input or discussion from their physicians or nurses. Physicians were asked to independently complete the NCICTC version 2.0 at the same time points as PNQ and HRQOL assessment to compare physician ratings against patients' grading. To ensure validity and accuracy of CIPN grading by the physicians, they were asked to make the NCI-CTC assessment after performing a required standardized neurological evaluation (tactile sensation, pain, and vibratory sensation) utilizing video instruction and an instruction leaflet developed by a neurologist and several oncologists [26].*"

c) *"The number and proportion of 300 patients completing the patient-reported CIPN and HRQOL questionnaires at baseline, before cycles 3, 5, and 7, at 7 months, and 1 year of study treatment were 295 (98 %), 295 (98 %), 279 (93 %), 270 (90 %), 260 (87 %), and 249 (83 %), respectively. The number (proportion) of physician-rated CIPN at the same time points was 293 (98 %), 287 (96 %), 281 (94 %), 269 (90 %), 256 (85 %), and 228 (76 %), respectively."* It doesn't say if there were any differences between those that answered and those that didn't.

## JBI CRITICAL APPRAISAL CHECKLIST FOR STUDIES REPORTING PREVALENCE DATA

Reviewer. Freja Løvendal Kruse, Date: 15/01/22

Author: Shivani S. Shinde, Year: 2016, Record Number: 14

| | Yes | No | Unclear | Not applicable |
|---|---|---|---|---|
| 100. Was the sample frame appropriate to address the target population? | X | □ | □ | □ |
| 101. Were study participants sampled in an appropriate way? | X | □ | □ | □ |
| 102. Was the sample size adequate? | □ | x | □ | □ |
| 103. Were the study subjects and the setting described in detail? | X | □ | □ | □ |
| 104. Was the data analysis conducted with sufficient coverage of the identified sample? | x | □ | □ | □ |
| 105. Were valid methods used for the identification of the condition? | x[a] | □ | □ | □ |
| 106. Was the condition measured in a standard, reliable way for all participants? | X | □ | □ | □ |
| 107. Was there appropriate statistical analysis? | x | □ | □ | □ |
| 108. Was the response rate adequate, and if not, was the low response rate managed appropriately? | □ | □ | x[b] | □ |

Overall appraisal: Include x Exclude □ Seek further info □

Comments (Including reason for exclusion):

a) EORTC-CIPN20 and CTCAE v.4.0 both validated PRO and CRO. See prior discribtion.

b) The number of patients went from 20 to 13 from baseline to 6 months follow up. It was not addressed why the patients were not followed up or if there were any differences between the groups.

## JBI CRITICAL APPRAISAL CHECKLIST FOR STUDIES REPORTING PREVALENCE DATA

Reviewer: Freja Løvendal Kruse, Date: 16/01/22

Author: Yuko Tanabe, Year: 2013, Record Number: 15

| | Yes | No | Unclear | Not applicable |
|---|---|---|---|---|
| 109. Was the sample frame appropriate to address the target population? | X | □ | □ | □ |
| 110. Were study participants sampled in an appropriate way? | X | □ | □ | □ |
| 111. Was the sample size adequate? | X | □ | □ | □ |
| 112. Were the study subjects and the setting described in detail? | X | □ | □ | □ |
| 113. Was the data analysis conducted with sufficient coverage of the identified sample? | x | □ | □ | □ |
| 114. Were valid methods used for the identification of the condition? | □ | X[a] | □ | □ |
| 115. Was the condition measured in a standard, reliable way for all participants? | □ | X[a] | □ | □ |
| 116. Was there appropriate statistical analysis? | X | □ | □ | □ |
| 117. Was the response rate adequate, and if not, was the low response rate managed appropriately? | □ | □ | □ | x[b] |

Overall appraisal: Include x Exclude □ Seek further info □

Comments (Including reason for exclusion):

a) CTCAE v 3.0, but done via chart review, which may give problems with judgement made without an option to clarify with the patient.

b) As the study was done through a retrospective chat review, respone rate, was not applicable.

## JBI CRITICAL APPRAISAL CHECKLIST FOR STUDIES REPORTING PREVALENCE DATA

Reviewer: Freja Løvendal Kruse, Date: 16/01/22

Author: Lisa M. Thornton, Year: 2008, Record Number: 16

| | Yes | No | Unclear | Not applicable |
|---|---|---|---|---|
| 118. Was the sample frame appropriate to address the target population? | X | □ | □ | □ |
| 119. Were study participants sampled in an appropriate way? | □ | x[c] | □ | □ |
| 120. Was the sample size adequate? | □ | x | □ | □ |
| 121. Were the study subjects and the setting described in detail? | x | □ | □ | □ |
| 122. Was the data analysis conducted with sufficient coverage of the identified sample? | x | □ | □ | □ |
| 123. Were valid methods used for the identification of the condition? | □ | x[a] | □ | □ |
| 124. Was the condition measured in a standard, reliable way for all participants? | □ | x[a] | □ | □ |
| 125. Was there appropriate statistical analysis? | □ | □ | x | □ |
| 126. Was the response rate adequate, and if not, was the low response rate managed appropriately? | □ | x[b] | □ | □ |

Overall appraisal: Include x Exclude □ Seek further info □

Comments (Including reason for exclusion):

a) A non-validated scale was used. Also parastesias was not specified to hands and feet: "*Unfortunately, the final item, parasthesia/numbness, referred to sensation at the surgical site rather than the periphery, which precluded our examination of the symptom in hands and feet. Greater than 90% of patients reported this symptom before receiving any chemotherapy. Therefore, it is not included in Figure 1.*"

*b)* From the article: *"At 60 months, follow-up data were available for 87 of 138 patients (63%). Of the 51 patients without 60-month data, 30 patients had developed recurrent disease or had died, and 21 patients had withdrawn from the study. The groups did not differ in the rates of recurrence, death, study withdrawal, or participation at 60 months (all P .205). Regarding the number of data points, there were 1216 completed assessments; 216 assessments were missing because of patient recurrence or death, 140 assessments were missing because of patient withdrawal from the study, and 94 assessments were missed by patients who continued participating in the study. All available data were used."*

*c)* Convinience sample.

## JBI CRITICAL APPRAISAL CHECKLIST FOR STUDIES REPORTING PREVALENCE DATA

Reviewer: Freja Løvendal Kruse, Date: 17/01/22

Author: Hannah C. Timmins, Year: 2021, Record Number: 17

| | Yes | No | Unclear | Not applicable |
|---|---|---|---|---|
| 127. Was the sample frame appropriate to address the target population? | X | □ | □ | □ |
| 128. Were study participants sampled in an appropriate way? | X | □ | □ | □ |
| 129. Was the sample size adequate? | □ | x | □ | □ |
| 130. Were the study subjects and the setting described in detail? | X | □ | □ | □ |
| 131. Was the data analysis conducted with sufficient coverage of the identified sample? | x | □ | □ | □ |
| 132. Were valid methods used for the identification of the condition? | X[a] | □ | □ | □ |
| 133. Was the condition measured in a standard, reliable way for all participants? | □ | x | □ | □ |
| 134. Was there appropriate statistical analysis? | □ | □ | x | □ |
| 135. Was the response rate adequate, and if not, was the low response rate managed appropriately? | x | □ | □ | □ |

Overall appraisal: Include x Exclude □ Seek further info □

Comments (Including reason for exclusion):

A) FACT-Ntx, TNSc, NCS and von frey filaments (upper limb) and two point discrimination (lower limb) (Touch-Test® Two-Point Discriminator). Validated assessments and a combination of modalities.
